# Pre-trained protein language model sheds new light on the prediction of Arabidopsis protein–protein interactions

**DOI:** 10.1186/s13007-023-01119-6

**Published:** 2023-12-07

**Authors:** Kewei Zhou, Chenping Lei, Jingyan Zheng, Yan Huang, Ziding Zhang

**Affiliations:** State Key Laboratory of Animal Biotech Breeding, College of Biological Sciences, China Agricultural University, Beijing, 100193 China

**Keywords:** Arabidopsis, Protein–protein interactions, Machine learning, Pre-trained language model, Natural language processing

## Abstract

**Background:**

Protein–protein interactions (PPIs) are heavily involved in many biological processes. Consequently, the identification of PPIs in the model plant Arabidopsis is of great significance to deeply understand plant growth and development, and then to promote the basic research of crop improvement. Although many experimental Arabidopsis PPIs have been determined currently, the known interactomic data of Arabidopsis is far from complete. In this context, developing effective machine learning models from existing PPI data to predict unknown Arabidopsis PPIs conveniently and rapidly is still urgently needed.

**Results:**

We used a large-scale pre-trained protein language model (pLM) called ESM-1b to convert protein sequences into high-dimensional vectors and then used them as the input of multilayer perceptron (MLP). To avoid the performance overestimation frequently occurring in PPI prediction, we employed stringent datasets to train and evaluate the predictive model. The results showed that the combination of ESM-1b and MLP (i.e., ESMAraPPI) achieved more accurate performance than the predictive models inferred from other pLMs or baseline sequence encoding schemes. In particular, the proposed ESMAraPPI yielded an AUPR value of 0.810 when tested on an independent test set where both proteins in each protein pair are unseen in the training dataset, suggesting its strong generalization and extrapolating ability. Moreover, the proposed ESMAraPPI model performed better than several state-of-the-art generic or plant-specific PPI predictors.

**Conclusion:**

Protein sequence embeddings from the pre-trained model ESM-1b contain rich protein semantic information. By combining with the MLP algorithm, ESM-1b revealed excellent performance in predicting Arabidopsis PPIs. We anticipate that the proposed predictive model (ESMAraPPI) can serve as a very competitive tool to accelerate the identification of Arabidopsis interactome.

**Supplementary Information:**

The online version contains supplementary material available at 10.1186/s13007-023-01119-6.

## Background

Protein–protein interactions (PPIs) are heavily involved in cellular biological processes, including signal transduction, transcriptional activation, and regulations of expression and metabolism [1]. Thus, it is critical to identify whether two proteins interact or not to help understand protein functions. Traditional experiments [e.g., isothermal titration calorimetry [2], pull-down assay [3], and surface plasmon resonance [4]] are low-throughput and time-consuming. With the development of high-throughput techniques, such as in vitro yeast two-hybrid screening [5] and affinity purification coupled with mass spectrometry [6], the identification of PPI data has been significantly accelerated, and the bioinformatics applications of PPI data have also been widely explored [7]. On the one hand, the experimental PPI data are often compiled as PPI interaction networks [8–10], and functionally unknown proteins in the networks can be annotated through network clustering and analysis [11–13]. On the other hand, the experimental PPI data can also be used to train PPI prediction models [14–16]. In this regard, machine learning is an increasingly popular computational method to learn data features deposited in known PPIs and build predictive models to predict unknown interactions.

Since protein interactions are mainly determined by their primary sequences, many efforts have focused on developing sequence-based PPI predictors. To build a machine learning model for PPI prediction, the key step is conducting feature engineering, which converts protein sequences into fixed-dimensional vectors. The frequently used sequence encoding schemes include amino acid composition (AAC), dipeptide composition (DPC), conjoint triad (CT), and composition of k-spaced amino acid pairs (CKSAAP). These descriptive representations are often combined with traditional machine learning methods, such as support vector machine (SVM) [17] and random forest (RF) [18], to develop effective PPI predictors. Regarding deep learning methods, primitive information without feature engineering can be used to extract more abstract representations. For instance, one-hot encoding and position-specific scoring matrix (PSSM) representation have been integrated with the framework of convolutional neural network (CNN) to achieve higher performance [19, 20].

By analogy to natural language, a protein sequence can be deemed a sentence, in which residue segments are regarded as words. Based on this hypothesis, natural language processing (NLP) methods have been used in protein representation. For instance, the typical word/sentence embedding techniques in NLP (e.g., word2vec and doc2vec) have been applied to protein sequence representations [21–23]. Although the word2vec/doc2vec models are either too shallow or trained with the corpus containing a limited number of existing proteins, they have revealed very promising results in many protein bioinformatics tasks. As one of the self-supervised language models, Transformer, released in 2017 by Google [24], solved the problem of memory capacity and processing speed. A typical Transformer is comprised of Attention modules focusing on vital information from global to local, and it often showed significant performance improvement when trained on large datasets [25]. Considering the advantages of Transformer in NLP, Rives et al. used this technique to generate a protein language model (pLM) for the purpose of protein sequence embeddings [26]. ESM is a deep Transformer language model trained on UniRef50, which can learn multi-scale representations, including biochemical properties, remote homology, and alignment within a protein family. Researchers have applied similar representation in different prediction tasks [27–31].

Large-scale identification of PPIs in the model plant Arabidopsis is of significance to decipher plant gene regulatory relationships, deeply understand plants’ growth and development, and promote the basic research of crop improvement and breeding. Although many known Arabidopsis PPIs have been stored in public databases, the Arabidopsis interactome remains incomplete. Thus, developing effective machine learning methods trained on existing PPI data to promptly predict unknown PPIs will accelerate the determination of Arabidopsis interactome data, reduce the experimental cost and provide new hints for plant functional genomics. To our best knowledge, however, the pLM-based embeddings have not been employed for predicting Arabidopsis PPIs. Here, we further explored the application of ESM representation in predicting Arabidopsis PPIs. Through a series of computational experiments, we observed that the combination of ESM-1b representation (one representative ESM model) with multilayer perceptron (MLP), termed ESMAraPPI, yielded more powerful performance than the predictive models inferred from other pLMs or conventional sequence features. In the meantime, the proposed model also revealed better performance than several generic or plant-specific PPI predictors.

## Results and discussion

### The computational framework and benchmarking datasets of ESMAraPPI

**Fig. 1:**
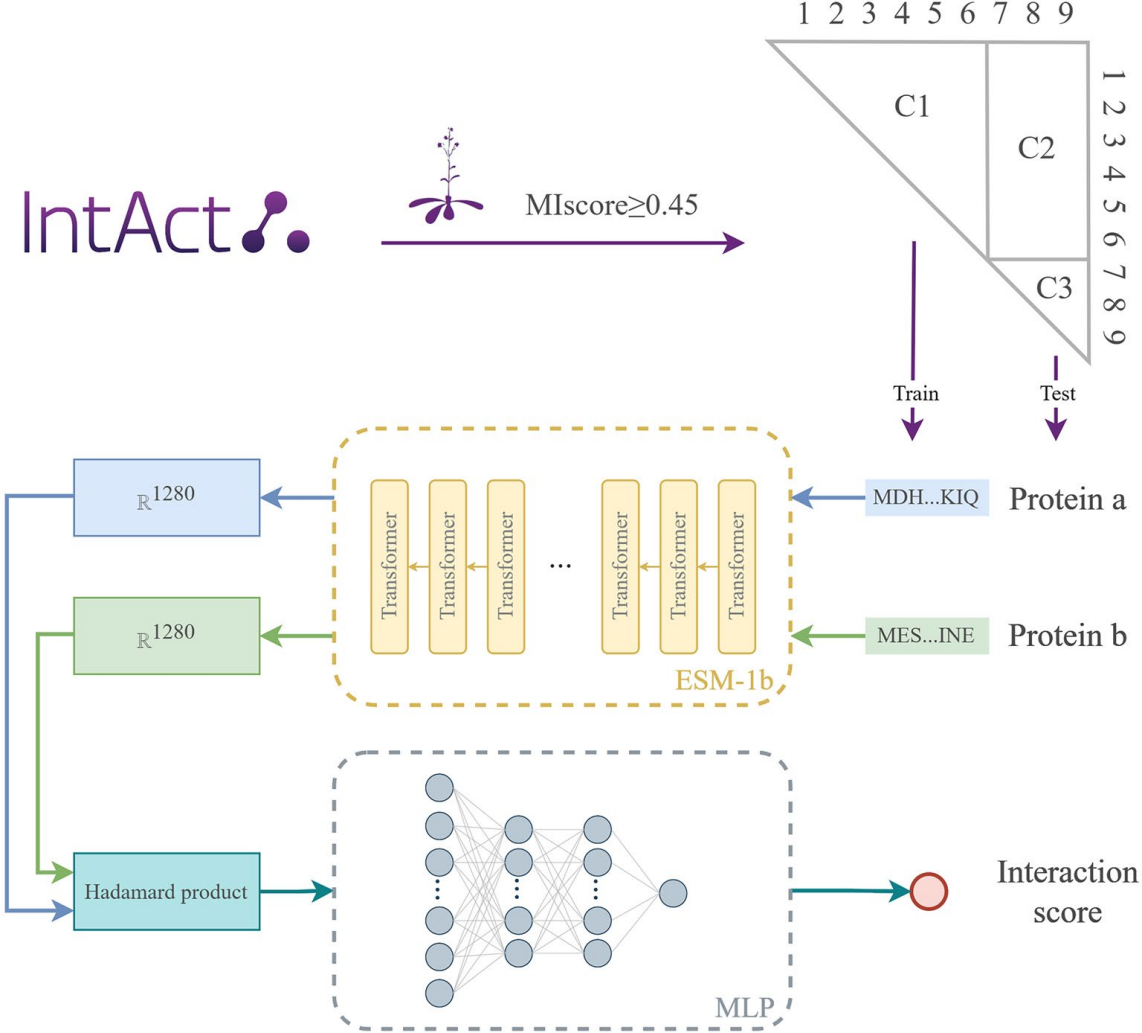
The schematic diagram of ESMAraPPI. Arabidopsis PPIs from the IntAct database with MIscore ≥ 0.45 were collected as positive samples. We also compiled 10 times negative samples to construct an original dataset. Then, we divided the original dataset into three datasets (i.e., C1, C2 and C3). C1 was the training dataset, while C2 and C3 were two independent test datasets. The representations of protein pair were extracted from ESM-1b, and Hadamard product was applied before inputting to a 4-layer MLP. The final output was an interaction score between 0 and 1 (a prediction score ≥ 0.5 corresponded to a positive interaction)

The flowchart of the proposed prediction method is illustrated in Fig. 1. To train and assess the performance of different PPI prediction models, we collected high-quality experimental Arabidopsis PPIs as positive samples. Additionally, we compiled negative training data by randomly selecting Arabidopsis protein pairs, and the ratio of positive to negative samples was set as 1:10. To train and evaluate model performance, we followed Park and Marcotte’s advice [32] to construct one training dataset (i.e., C1) and two independent test sets (i.e., C2 and C3) (Fig. 1). Specifically, only one protein in each pair from C2 is allowed to be appeared in C1, whereas both proteins in each pair from C3 are unseen in C1. The representation of a protein was extracted from ESM-1b directly, which resulted in a feature vector of 1280 dimensionality. Since the PPI prediction is a pair-input problem, Hadamard product was applied before inputting representations of protein pairs to a 4-layer MLP for model training. More details about the dataset preparation, pLM feature vector construction, and machine learning algorithm implementation are available in the Methods section.

### ESM-1b coupled with MLP performed best in predicting Arabidopsis PPIs

**Fig. 2:**
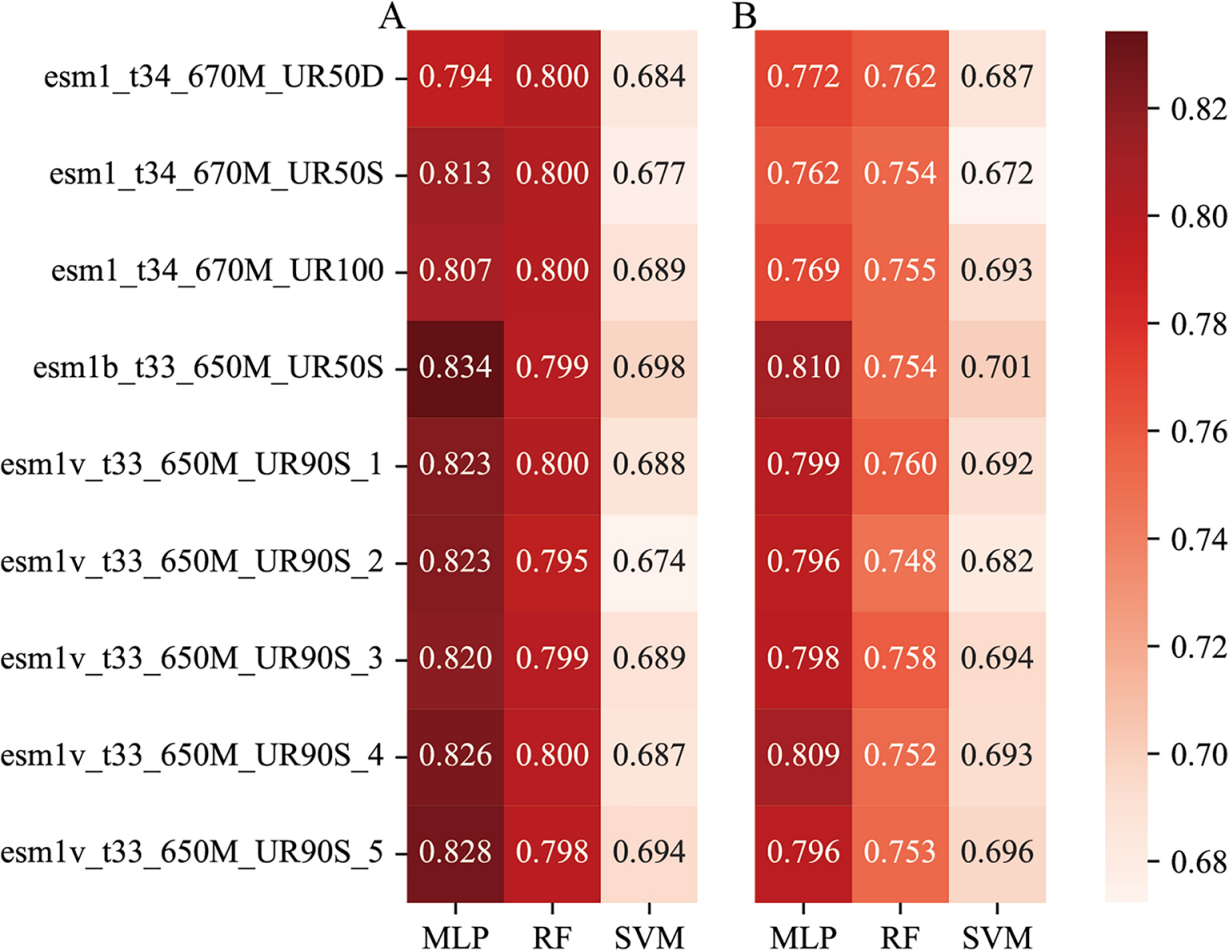
AUPR values of combinations between nine pLMs from ESM and three machine learning algorithms. Of the different ESM models, ESM-1v was fine-tuned for predicting variant effects and contained five models with different random seeds. ESM-1b differs from ESM-1 mainly in higher learning rate, dropout after word embedding, learned positional embeddings, final layer norm before the output, and tied input/output word embedding. **A** The results from the independent dataset C2 where only one protein in each pair appeared in the training dataset (i.e., C1), while **B** corresponds to the results from the independent dataset C3 where no protein in each pair appeared in the training dataset (i.e., C1)

Nine different pLMs from ESM embed each protein sequence to a vector of 1280 dimensionality. We combined these pLMs with three machine learning algorithms (MLP, RF and SVM) to seek the best combination. The 4-layer MLP computational framework, which contains 1024, 512, 128, and 16 nodes, was optimally selected. Concerning RF and SVM, the corresponding parameters were optimized through grid search. Considering that positives and negative samples are highly imbalanced in this work, we mainly quantified the performance by plotting the precision–recall (PR) curve and calculating the corresponding area under the PR curve (AUPR). As shown in Fig. 2, MLP-based models yielded the highest AUPR values, followed by RF- and SVM-based models. In particular, the combination of MLP and ESM-1b (i.e., esm1b_t33_650M_UR50S) achieved the best performance (AUPR = 0.834 on C2 and 0.810 on C3). To supplement the AUPR-based assessment, we also plotted the receiver operating characteristic (ROC) curve and calculated the corresponding area under the ROC curve (AUROC) for each combination. Again, the MLP and ESM-1b combination resulted in the largest AUROC value (Additional file 1: Fig. S1).

**Fig. 3:**
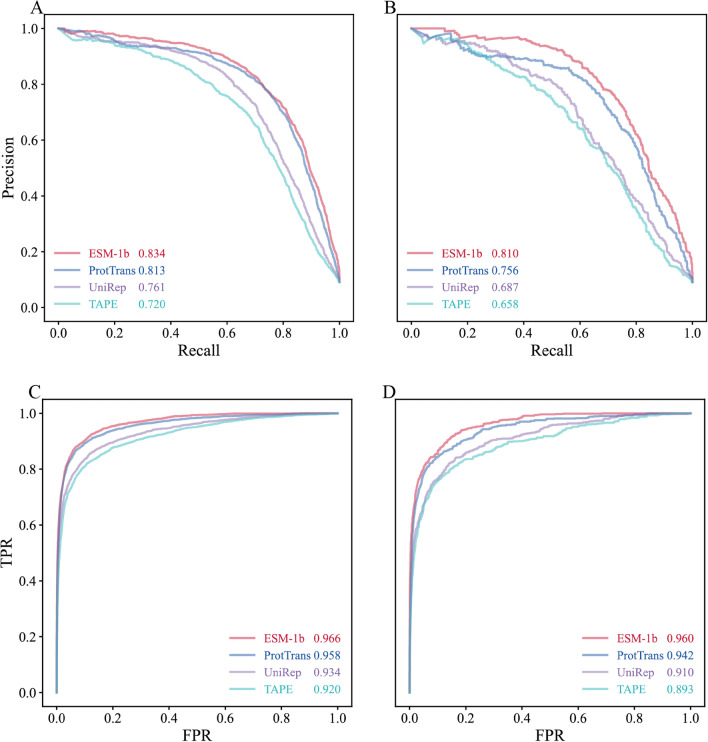
PR and ROC curves of the predictive models from four pLMs in combination with MLP. **A** plots the PR curves on the independent test set C2, while **B** plots the PR curves on the independent test set C3. Parameters in the legends of **A** and **B** denote the corresponding AUPR values. **C** plots the ROC curves on the independent test set C2, while **D** plots the ROC curves on the independent test set C3. Parameters in the legends of **C** and **D** denote the corresponding AUROC values

We further compared ESM-1b with three pLMs, ProtTrans, UniRep, and TAPE. Note that these three pLMs were trained using different training strategies from ESM-1b. Trained on data from UniRef and BFD covering up to 393 billion amino acids, an auto-encoder model (ProtT5-XL-U50) from ProtTrans for the first time outperformed existing methods without the need of multiple sequence alignments (MSAs) or evolutionary information in secondary structure prediction [33]. UniRep was based on multiplicative LSTM (mLSTM) and was trained on UniRef50 [34]. It was found that the amino-acid embeddings learned by UniRep contained physiochemically meaningful clusters. TAPE was a small Transformer trained on UniRef50 [35], which embedded each protein sequence to a vector of 768 and achieved comparable performance with UniRep on protein fluorescence and stability prediction. Of the three machine learning algorithms under investigation, the MLP algorithm again achieved the best performance in combination with these three pLMs judged by AUPRC or AUROC values. Interestingly, ESM-1b also outperformed three other pLMs in the computational framework of MLP (Fig. 3). We further compared ESM-1b with two baseline sequence encoding schemes (i.e., AAC and DPC). AAC stands for the compositions of each amino acid in the whole protein sequence, which transforms a protein into a vector of 20 dimensionality. DPC represents the compositions of two continuous amino acids in the whole protein sequence, which was used to convert a protein into a vector of 400 dimensionality. As shown in Additional file 1: Table S1, the combination of AAC and SVM seems to be optimal (AUPR = 0.519 on C2 and 0.481 on C3; AUROC = 0.852 on C2 and 0.824 on C3), while the combination of DPC and RF achieves the best performance (AUPR = 0.646 on C2 and 0.564 on C3; AUROC = 0.884 on C2 and 0.845 on C3). Comparatively, the optimal performance of these two traditional encoding schemes is much inferior to that of ESM-1b.

### Comparison to existing generic PPI prediction methods

We compared our method with four generic PPI prediction methods, including three sequence-based methods [i.e., D-SCRIPT [16], RAPPPID [36], and PIPR [37]] and one structure-based method [i.e., TAGPPI [38]]. D-SCRIPT first applied a pre-trained model to generate structurally informative feature representations of proteins, and then estimates an interaction probability of protein pairs based on these features. RAPPPID is a deep learning-based PPI predictor implemented through a twin averaged weight-dropped LSTM network employing multiple regularization methods in the training step to learn generalized weights. When tested on stringent PPI datasets containing proteins unseen in the training dataset, it reveals excellent performance. PIPR is a sequence-based PPI predictor combining pre-trained amino acid embeddings with a Siamese recurrent convolutional neural network (RCNN) architecture. TAGPPI is an end-to-end computational framework for PPI prediction, in which multi-dimensional features by employing 1D convolution operation on protein sequences and graph learning method on contact maps constructed from AlphaFold2 are considered.

**Fig. 4:**
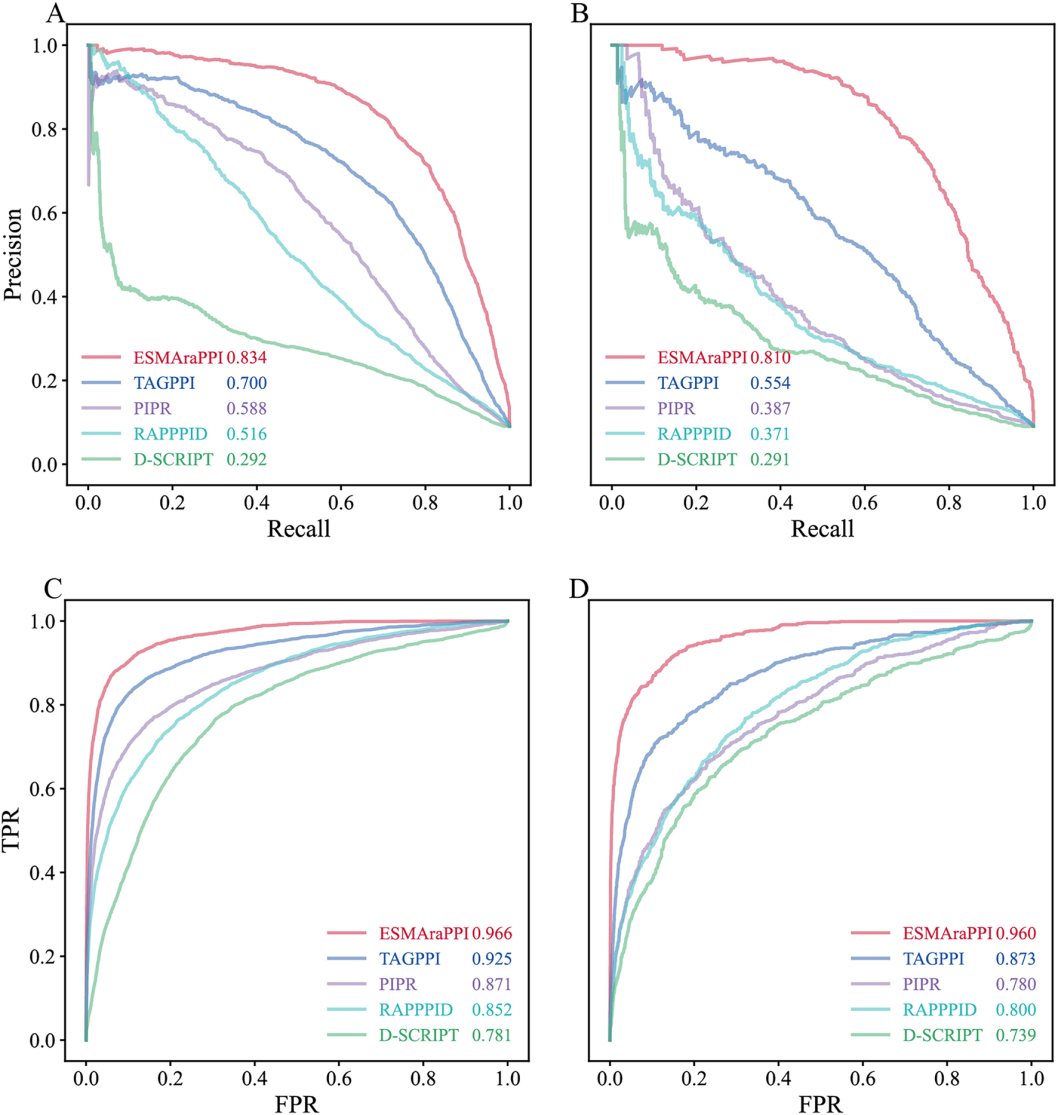
PR and ROC curves of ESMAraPPI and four existing generic PPI predictors in predicting Arabidopsis PPIs. **A** plots the PR curves on the independent test set C2, while **B** plots the PR curves on the independent test set C3. Parameters in the legends of **A** and **B** denote the corresponding AUPR values. **C** plots the ROC curves on the independent test set C2, while **D** plots the ROC curves on the independent test set C3. Parameters in the legends of **C** and **D** denote the corresponding AUROC values

We downloaded the source codes of PIPR, D-SCRIPT, RAPPPID, and TAGPPI and retrained the corresponding predictive models using the C1 dataset. Moreover, we tested their performance on our two independent datasets (C2 and C3). As shown in Fig. 4, the proposed ESMAraPPI considerably outperformed the four existing PPI predictors in terms of AUPRC or AUROC values. In particular, the proposed ESMAraPPI reveals robust performance on the C3 test set. For the three existing sequence-based methods, the performance of PIPR is ranked as the best, followed by RAPPPID and D-SCRIPT. When tested on the C3 test set, the performance ranking remains the same, but the performance of PIPR and RAPPPID dropped rapidly. Considering the predicted protein structural information used in PPI prediction, TAGPPI considerably surpassed these three pure sequence-based models on both C2 and C3, although it also dropped sharply on C3.

In addition to the better performance of ESM-1b embedding with simple MLP, its computational efficiency is also high. In either the model training or prediction steps, ESMAraPPI showed more rapid computational speed (Table 1).

**Table 1:** Computational time required in different methods^a^

	ESM-1b + MLP	TAGPPI	RAPPPID	PIPR	D-SCRIPT
Training epoch	40	10	20	20	10
Total training time	56 s	9.29 h	1.12 h	700 s	7.22 h
Total predicting time^b^	0.1 s	583 s	18 s	5 s	82 s

^a^All the training and prediction procedures were processed on a high-performance computer with 20 cores CPU, 256G RAM, and Tesla V100 GPU

^b^ Total predicting time means the computational time required for processing the C3 test dataset

### Comparison to existing Arabidopsis PPI prediction methods

We compared the proposed ESMAraPPI with two existing Arabidopsis PPI prediction methods [i.e., AraPPINet [39] and DeepAraPPI [40]]. AraPPINet was inferred from three-dimensional structures and functional evidence, encompassing 316,747 high-confidence interactions among 12,574 proteins. It exhibited high predictive power for discovering protein interactions at a 50% true positive rate. To allow for a fair comparison between our model and AraPPINet, we submitted our test datasets (C2 and C3) directly to their web server, and the default threshold (0.5) reported by AraPPINet was also used to distinguish interactions and non-interactions. Since AraPPINet did not release the predictions at different threshold values, we were not able to compare ESMAraPPI and AraPPINet through AUPRC or AUROC values. Thus, the routine measurements, such as Accuracy, Matthews correlation coefficient (MCC), Recall [i.e., true positive rate (TPR)], Specificity [i.e., 1-false positive rate (FPR)] and Precision, were employed for performance comparison (Table 2). Judged by the MCC value, which is a more comprehensive measurement than the other parameters, ESMAraPPI outperforms AraPPINet in both C2 and C3 datasets (Table 2).

**Table 2:** Comparison of ESMAraPPI and AraPPINet on the C2 and C3 test sets

Methods	C2					C3				
	Accuracy	Specificity	MCC	Recall	Precision	Accuracy	Specificity	MCC	Recall	Precision
ESMAraPPI	0.957	0.994	0.708	0.589	0.901	0.954	0.994	0.688	0.557	0.902
AraPPINet	0.939	0.999	0.551	0.337	0.966	0.937	0.999	0.534	0.318	0.966

We finally compared ESMAraPPI with DeepAraPPI, which was recently developed in our team. As an integrative Arabidopsis PPI prediction method, DeepAraPPI comprises three individual predictors, (i) a word2vec encoding-based Siamese RCNN model, (ii) a Domain2vec encoding-based MLP model, and (iii) a GO2vec encoding-based MLP model [40]. The final DeepAraPPI model combined the prediction results of the three individual predictors through a Logistic regression model. We also tested DeepAraPPI on our datasets. As shown in Table 3, ESMAraPPI outperformed the individual predictors of DeepAraPPI (i.e., RCNN, Domain2vec, and Go2vec) in both test sets (C2 and C3). With respect to C3, ESMAraPPI surpassed the final DeepAraPPI model, which means our new method was more competitive and will be more reliable in practical applications.

**Table 3:** AUPR and AUROC values of DeepAraPPI and ESMAraPPI on the C2 and C3 test sets^a^

Method	AUPR		AUROC	
	C2	C3	C2	C3
DeepAraPPI_RCNN	0.541	0.331	0.852	0.778
DeepAraPPI_Domain2vec	0.706	0.639	0.884	0.845
DeepAraPPI_Go2vec	0.771	0.709	0.942	0.917
DeepAraPPI	**0.871**	0.785	**0.978**	0.944
ESMAraPPI	0.824	**0.810**	0.966	**0.960**

^a^ Figure in bold font indicates the corresponding model achieved the maximal AUPR and AUROC value

## Case study

To explore the real application of ESMAraPPI, we provided a case study related to the interaction prediction of two proteins (BIN2 and SOS2) involved in the salt overly sensitive (SOS) pathway. In 2020, Li et al. showed that BIN2 functions as a negative regulator of primary root growth under salt stress by phosphorylating and inhibiting SOS2 [41]. It should be emphasized that the interaction between BIN2 and SOS2 was consistently determined by the yeast two-hybrid assay, the split-LUC assay and the BiFC assay in Li et al.’s work, which has not been included in any public database. Using the ESMAraPPI model, BIN2 and SOS2 were predicted to interact (prediction score = 0.592), indicating the proposed method has practical application in predicting Arabidopsis PPIs.

## Conclusion

In this work, we found that sequence representations directly generated by large-scale pre-trained pLMs without any further feature engineering can be successfully used to develop machine learning-based Arabidopsis PPI predictors. We have shown that the proposed ESMAraPPI (i.e., ESM-1b + MLP) model yielded a highly accurate performance in predicting Arabidopsis PPIs. On the one hand, it achieved dramatic performance improvement in comparison to the models inferred from baseline sequence encoding schemes. On the other hand, it also revealed better performance than several state-of-the-art generic or plant-specific PPI predictors. The success of ESMAraPPI should be ascribed to the fact that the large-scale pre-trained pLMs can capture rich semantic information regarding protein sequence-structure-evolution relationships. To facilitate the research community, we have made all our codes and datasets freely available at https://github.com/keiwo/ESMAraPPI. We believe that the application of pLMs in protein sequence representation is providing a very promising way to deal with feature engineering in PPI prediction.

## Methods

### Data collection and preprocessing

Experimental Arabidopsis PPIs were first downloaded from IntAct (https://www.ebi.ac.uk/intact/home), and only PPIs with the type of direct interaction or physical association were further retained. Moreover, PPIs with MIscore < 0.45 were removed. Finally, we obtained 7729 PPIs, which are regarded as positive samples in this work. To construct negative samples, we first removed proteins in positive samples from the complete Arabidopsis protein list, and the remaining Arabidopsis proteins sharing ≥ 40% sequence identity with proteins in positive samples were further filtered out. Then, we removed redundant proteins by applying a sequence identity cut-off of 40%, and 8382 proteins were retained. After that, we obtained a protein list by mixing these 8382 proteins and proteins in positive samples, which were used to construct negative samples through random pairing. By controlling the ratio of positive and negative samples as 1:10, 77,290 random protein pairs that were not experimentally identified as PPIs were selected as negative samples. Finally, an original dataset containing 7729 positive samples (i.e., PPIs) and 77,290 negative samples (i.e., non-PPIs) was compiled in this work. To conduct model training and evaluation, we followed Park and Marcotte’s advice to divide the original dataset into three datasets (i.e., C1, C2, and C3). C1 was the train dataset, while C2 and C3 were two independent test datasets. More details about the sizes of the three datasets are listed in Table 4.

**Table 4:** Statistics of the C1, C2 and C3 datasets

Dataset	#positive samples	#proteins involved in positive samples	#negative samples	#proteins involved in negative samples
C1	3519	1415	35,190	7068
C2	3404	1781	34,040	10,586
C3	806	551	8060	3534

### Protein representation

The ESM models are available at https://github.com/facebookresearch/esm/tree/v1.0.2. There are 13 models in the ESM version we used. Of them, esm_msa1_t12_100M_UR50S and esm_msa1b_t12_100M_UR50S require extra MSAs as input, which were not further considered. In the remaining 11 models, nine models encode each protein sequence into a vector of 1280 dimensionality, which were chosen for further investigation. We downloaded these nine pre-trained ESM models and followed the ESM authors’ instructions to run them locally. After extracting the final layer’s hidden parameters, the matrix was averaged on the first dimension to generate 1280 features for each sequence. The ProtTrans model is available at https://github.com/agemagician/ProtTrans. We downloaded prot_t5_xl_uniref50 (ProtT5), which converted each protein sequence to a vector of 1024 dimensionality. The TAPE and UniRep models are available at https://github.com/songlab-cal/tape. Similarly, we downloaded TAPE and UniRep that embed each protein sequence to a vector of 1900 and 768 dimensionality, respectively.

### Machine learning algorithms

#### Multilayer perceptron (MLP)

Through the PyTorch machine learning framework, we implemented a 4-layer MLP, which contains 1024, 512, 128, and 16 nodes. To avoid the order bias from protein pairs, the Hadamard product of two protein features, rather than their concatenation, was used as model input. The sigmoid function was applied to the final output to yield a prediction score between 0 and 1 (a prediction score ≥ 0.5 corresponded to a positive interaction). Then, the binary cross entropy (BCE) loss function was implemented.

#### Support vector machine (SVM)

We implemented SVM based on the sklearn package in Python, and the parameters were optimized by grid search. The kernel function was set as 'rbf', the regularization parameter was set to 1, and the kernel coefficient was set as 'scale'. The other parameters were set as default. The model input was Hadamard product of two protein features. A prediction score ≥ 0.5 was thought to be interaction.

#### Random forest (RF)

We implemented RF based on the sklearn package in Python. The parameters were optimized by grid search. The n_estimators was set as '100', and max_depth was set as 'None'. The model input was Hadamard product of two protein features. A prediction score ≥ 0.5 was thought to be interaction.

### Performance evaluations

Accuracy, Specificity, Precision, Recall, and MCC were used to evaluate the prediction performance. These parameters are defined as follows:

$$Accuracy=\frac{TP+TN}{TP+FP+TN+FN}$$

$$Specificity=1-FPR=\frac{TN}{TN+FP}$$

$$Precision=\frac{TP}{TP+FP}$$

$$Recall=TPR=\frac{TP}{TP+FN}$$

$$MCC=\frac{TP\times TN-FP\times FN}{\sqrt{(TP+FP)\times (TP+FN)\times (TN+FP)\times (TN+FN)}}$$

where TP, TN, FP, and FN represent the numbers of true positives, true negatives, false positives, and false negatives, respectively. To provide a comprehensive performance assessment for each predictive model, the PR curve was plotted, and the AUPRC value was also calculated to quantify the performance. In the meantime, the ROC curve, which plots the TPR value against the FPR value at different thresholds, and the corresponding AUROC value were also employed for performance assessment.

### Supplementary Information

**Additional file 1. **

## Data Availability

The codes and datasets supporting the conclusions of this article are available in the GitHub repository, https://github.com/keiwo/ESMAraPPI.
