# Supplementary material for: Pre-trained protein language model sheds new light on the prediction of Arabidopsis protein–protein interactions
Source: Plant Methods. 2023 Dec 7;19:141. doi: 10.1186/s13007-023-01119-6 (PMC10704805; doi:10.1186/s13007-023-01119-6)
Supplement: Supplementary file 1 — Additional file 1. [file 13007_2023_1119_MOESM1_ESM.docx]

**Supplementary material**

**Pre-trained protein language model sheds new light on the prediction of Arabidopsis protein-protein interactions**

Kewei Zhou, Chenping Lei, Jingyan Zheng, Yan Huang, Ziding Zhang*

*State Key Laboratory of Farm Animal Biotech Breeding, College of Biological Sciences, China Agricultural University, Beijing 100193, China*

^*^Corresponding author: Dr. Ziding Zhang, E-mail: zidingzhang@cau.edu.cn

***This supplementary material contains Table S1 and Fig. S1.***

Table S1 AUPR and AUROC values of ESMAraPPI and two baseline sequence encoding schemes on the C2 and C3 test datasets^a^.

| Method | AUPR | |  | AUROC | |
| --- | --- | --- | --- | --- | --- |
|  | C2 | C3 |  | C2 | C3 |
| ESMAraPPI | **0.824** | **0.810** |  | **0.966** | **0.960** |
| AAC+SVM | 0.519 | 0.481 |  | 0.852 | 0.824 |
| AAC+RF | 0.563 | 0.479 |  | 0.856 | 0.817 |
| DPC+SVM | 0.615 | 0.543 |  | 0.868 | 0.832 |
| DPC+RF | 0.646 | 0.564 |  | 0.884 | 0.845 |

^a^ Figure in bold font indicates the corresponding model achieved the maximal AUPR and AUROC value.


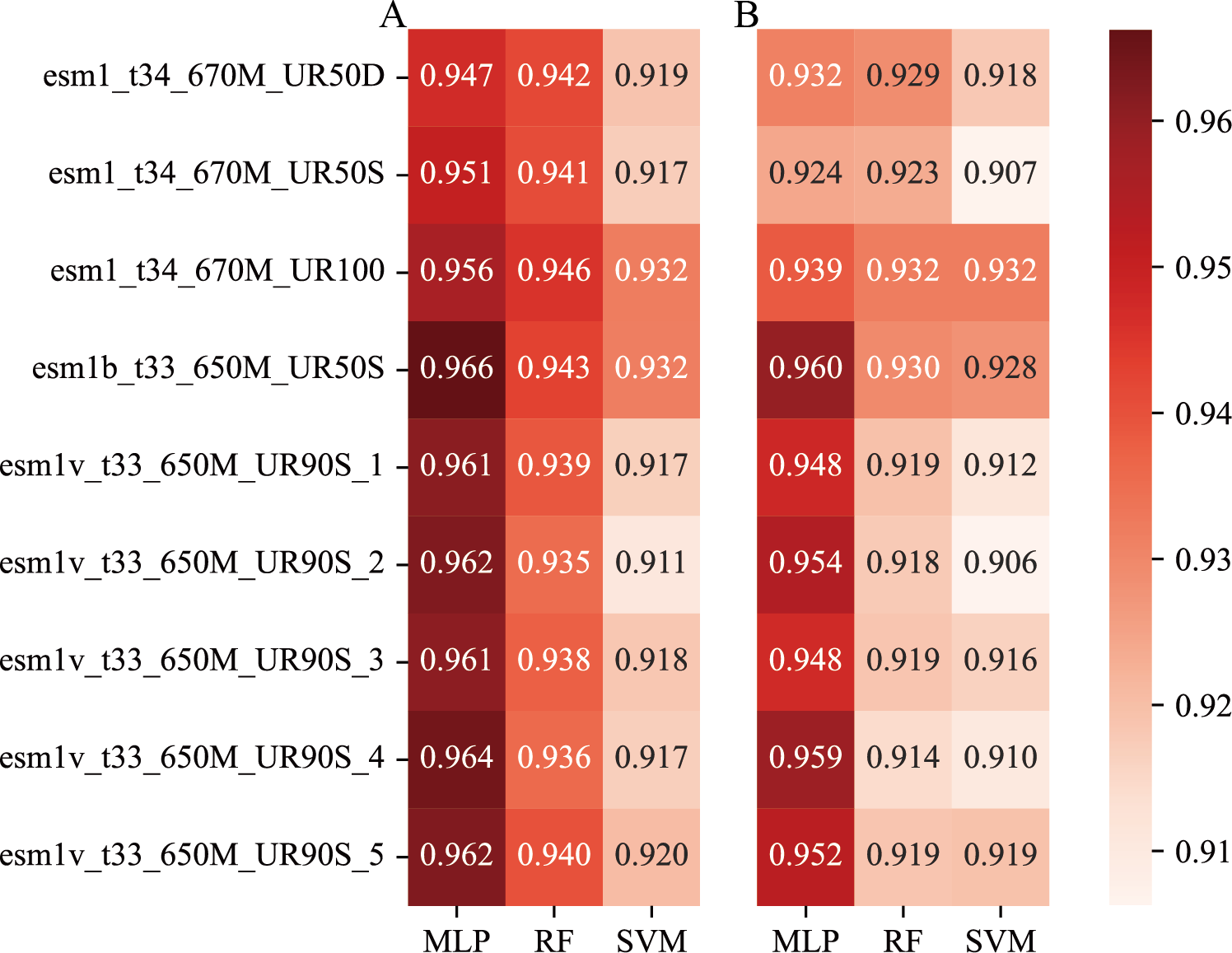


**Fig. S1 AUROC values of combinations between nine pLMs from ESM and three machine learning algorithms.** Of the different ESM models, ESM-1v was fine-tuned for predicting variant effects and contained five models with different random seeds. ESM-1b differs from ESM-1 mainly in higher learning rate, dropout after word embedding, learned positional embeddings, final layer norm before the output, and tied input/output word embedding. Panel A represents the results from the independent dataset C2 where only one protein in each pair appeared in the train dataset (i.e., C1), while panel B corresponds to the results from the independent dataset C3 where no protein in each pair appeared in the training dataset (i.e., C1).
